# A computer‐aided diagnosis tool in prostate cancer patients with biochemical recurrence using 18F‐PSMA PET/CT imaging

**DOI:** 10.1002/mp.70572

**Published:** 2026-07-07

**Authors:** Ioanna Stamouli, Ilias Gatos, Theodoros Kalathas, Stavros Tsantis, Lydia‐Aggeliki Zoglopitou, Paraskevi F. Katsakiori, Nikolaos Papathanasiou, Anna Makridou, Vassiliki Chatzipavlidou, John D. Hazle, George C. Kagadis

**Affiliations:** ^1^ 3DMI Research Group, Department of Medical Physics, School of Medicine University of Patras Rion Greece; ^2^ Nuclear Medicine Department Cancer Hospital of Thessaloniki Theagenio Thessaloniki Greece; ^3^ Department of Nuclear Medicine School of Medicine University of Patras Rion Greece; ^4^ Department of Imaging Physics The University of Texas MD Anderson Cancer Center Houston Texas USA

**Keywords:** CAD, prostate cancer, PSMA

## Abstract

**Background:**

Prostate cancer (PCa) patients frequently experience biochemical recurrence (BCR) following definitive primary treatment. Although fluorine‐18–labeled prostate‐specific membrane antigen positron emission tomography/computed tomography (^1^ ^8^F‐PSMA PET/CT) is an imaging modality that is highly sensitive for BCR, false‐positive findings owing to benign nonspecific uptake complicate diagnosis. Existing artificial intelligence (AI) tools have attempted to address this challenge but are often limited by their reliance on ground‐truth labels derived from expert visual interpretation; thus, these tools reproduce expert opinion rather than confirming disease status.

**Purpose:**

The purpose of this study was to develop a computer‐aided diagnosis (CAD) system for classifying benign and malignant findings on ^1^ ^8^F‐PSMA PET/CT imaging in patients with PCa and BCR. Post‐therapy imaging follow‐up was used as an objective reference standard for malignancy.

**Methods:**

A dataset of 69 patients with PCa and BCR who underwent ^1^ ^8^F‐PSMA PET/CT imaging was used to develop a CAD. The system was evaluated under two classification schemes based on different ground truths: Task 1 used post‐therapy imaging follow‐up as the objective reference standard for malignant findings (in a subset of 45 patients), whereas task 2 relied on expert visual interpretation at baseline. In total, after data augmentation and filtering, 334 findings were analyzed for task 1, and 467 findings were analyzed for task 2. Suspicious findings were manually segmented using LifeX software (version 25.06.1). One‐dimensional intensity profiles were extracted along the *x*‐, *y*‐, and *z*‐axes at the maximum intensity voxel of each finding, from which profile‐based and Gaussian‐fit features were derived. The intensity profiles were used directly as inputs to a multilayer perceptron (MLP) classifier and were also used to extract profile‐ and Gaussian‐fit–based features to train a random forest (RF) classifier using feature‐importance analysis. The final model incorporated a stacking ensemble architecture combining the MLP and RF base models; logistic regression was the meta‐classifier. The model was trained and evaluated using stratified 10‐fold cross‐validation. In each fold, 90% of the findings were assigned to the training set for model development, including feature selection, and the remaining 10% were held out as an independent test set for performance evaluation. Within each training set, five‐fold internal cross‐validation was used as the validation procedure for feature selection and stacking.

**Results:**

For task 1, the stacking ensemble model achieved an accuracy of 92.5% (SD, 3.5%), sensitivity of 92.3% (SD, 4.6%), specificity of 93.0% (SD, 9.2%), and an area under the receiver operating characteristic curve (AUROC) of 0.97. For task 2, performance was similar: accuracy, 91.7% (SD, 5.2%); sensitivity, 93.8% (SD, 4.5%); specificity, 85.8% (SD, 8.6%); and AUROC, 0.97. Feature‐importance analysis revealed that raw intensity magnitude and spatial gradients along the *x*‐axis were the most discriminative features for classification.

**Conclusions:**

The proposed CAD system achieved highly accurate classification of ^1^ ^8^F‐PSMA PET/CT findings, leveraging post‐therapy imaging follow‐up as a more objective reference standard for identifying malignancy than expert‐based visual interpretation alone. The system achieved high and consistent performance across both the follow‐up–based and expert‐based labeling tasks. The integration of this tool into clinical workflow could improve diagnostic confidence and support the personalized management of PCa patients with BCR.

## 1 INTRODUCTION

Prostate cancer (PCa) is the second leading cause of cancer‐related mortality in men in the United States, with approximately 310 000 new cases and 35 000 deaths projected in 2025.^1^ Many patients develop biochemical recurrence (BCR) after treatment: 27%–53% of men experienced increased prostate‐specific antigen (PSA) levels after radical prostatectomy or radiotherapy.^2^ Following prostatectomy, BCR is defined as a PSA level above 0.2 ng/mL that continues to rise, whereas after radiotherapy, BCR is defined as a PSA level increase of more than 2 ng/mL above the post‐treatment nadir.^3^, ^4^ PSA elevation indicates recurrence but does not localize the extent of disease, highlighting the critical role of imaging in guiding treatment decisions.^2^

Fluorine‐18–labeled prostate‐specific membrane antigen positron emission tomography/computed tomography (^1^ ^8^F‐PSMA PET/CT) is the preferred imaging modality for BCR, offering superior sensitivity and specificity over conventional CT, bone scintigraphy, and earlier tracers such as ^1^ ^8^F‐choline.^5^, ^6^ ^1^ ^8^F‐PSMA‐1007 offers advantages over other tracers such as gallium‐68–labeled PSMA, including higher spatial resolution and minimal renal excretion, that reduces urinary background activity and aids the interpretation of lesions near the bladder and ureters.^7^, ^8^, ^9^

Despite its high sensitivity, ^1^ ^8^F‐PSMA PET/CT is susceptible to false‐positive findings. ^1^ ^8^F‐PSMA‐1007 has been associated with a relatively high incidence of nonspecific bone uptake without morphologic correlation, which may be mistaken for metastases, particularly in patients with low or undetectable PSA levels.^10^ Benign conditions such as hemangiomas, fractures, and fibrous dysplasia are also associated with elevated PSMA expression.^11^ In clinical practice, the histopathologic confirmation of every suspicious finding is not feasible; this issue impedes the establishment of a reliable ground truth, and the distinction between malignant and false‐positive foci therefore has remained unresolved in many studies.^10^, ^12^ Better reference standards are therefore needed to support the accurate differentiation between benign and malignant uptake.^13^, ^14^

Although artificial intelligence (AI) approaches have been proposed for PSMA PET/CT analysis, most approaches rely on expert visual interpretation rather than histopathologic confirmation as the ground truth, meaning trained models effectively learn to reproduce expert opinion rather than confirmed disease status.^15^, ^16^, ^17^ Erle et al.,^15^ Li et al.,^16^ and Moazemi et al.^17^ reported high area under the receiver operating characteristic curve (AUROC) values (0.97–0.98) using machine‐learning or deep‐learning frameworks but acknowledged expert‐based labeling as a key limitation of their approaches.^15^, ^16^, ^17^ Zhao et al.^18^ attempted to address this limitation by incorporating pathologic or imaging follow‐up as a reference standard but achieved only moderate model performance (accuracy, 0.78; AUROC, 0.79), underscoring the need for improved labeling strategies.

The purpose of this study was to develop a computer‐aided diagnosis (CAD) system for classifying benign and malignant findings on ^1^ ^8^F‐PSMA PET/CT imaging in patients with PCa and BCR. Post‐therapy PET/CT follow‐up examinations from patients who exhibited a response to treatment were used to retrospectively confirm whether suspicious findings were malignant. Findings showing a visible reduction in tumor size and/or ^1^ ^8^F‐PSMA uptake on follow‐up PET/CT compared with baseline were considered malignant, providing greater diagnostic confidence than single‐time‐point expert interpretation alone. Following pre‐processing and 3‐dimensional segmentation of suspicious findings, a supervised classification approach was introduced that, to our knowledge, used features extracted from one‐dimensional intensity profiles along the *x*‐, *y*‐, and *z*‐axes centered on the maximum uptake voxel of each finding for the first time. Profile‐based and Gaussian‐fit–based features were used as inputs for a random forest (RF) classifier, and raw intensity profiles were fed to a multilayer perceptron (MLP) classifier; the outputs of both classifiers were combined in a stacking ensemble architecture. This approach trained the system using a robust labeling strategy, providing more reliable classification and diagnostic confidence than single‐time‐point expert interpretation alone.

## 2 METHODS

### 2.1 Ethical approval

This study was conducted according to the ethical guidelines of the Declaration of Helsinki. Furthermore, Institutional Review Board approval was received for this study.

### 2.2 Patients

The clinical dataset included 69 men (mean age 71 (SD, 8) years) with PCa who experienced BCR. All patients underwent baseline ^18^F‐PSMA‐1007 PET/CT imaging at Theagenio Cancer Hospital of Thessaloniki between May 2019 and December 2024. Post‐treatment follow‐up PET/CT imaging was available for 45 patients (5–19 months after baseline imaging), but 24 patients did not undergo follow‐up imaging.

Patients were eligible for inclusion if they met all of the following criteria: (i) pathologically or clinically confirmed PCa, (ii) BCR following definitive primary treatment, regardless of the type of curative therapy received, (iii) clinical referral for ^1^ ^8^F‐PSMA‐1007 PET/CT imaging performed between May 2019 and December 2024, and (iv) age ≥ 18 years. Patients were excluded if they had a concurrent active second malignancy at the time of imaging.

### 2.3 Imaging acquisition and interpretation

All patients underwent ^1^ ^8^F‐PSMA‐1007 PET/CT imaging from the vertex to the mid‐thighs on a Discovery 710 PET/CT system (GE Healthcare) at baseline and follow‐up. Image acquisition started 60 min after the intravenous administration of 3–4 MBq/kg body weight of ^18^F‐PSMA‐1007. Each acquisition consisted of 5–6 bed positions, with an acquisition time of 2–3 min per bed position. The images were reconstructed using a three‐dimensional ordered‐subset expectation maximization algorithm with time‐of‐flight information, utilizing 2 iterations and 24 subsets with no post‐reconstruction filtering. The CT acquisition was set to 120 kVp in helical mode with a slice thickness of 3.75 mm.

All baseline and follow‐up ^1^ ^8^F‐PSMA‐1007 PET/CT examinations were independently reviewed by an experienced nuclear medicine physician. Image interpretation followed the European Association of Nuclear Medicine procedure guidelines for PSMA PET/CT imaging, accounting for tracer uptake intensity, anatomic correlation on CT, and lesion morphology.^19^ For each examination, the nuclear medicine physician classified the findings as physiologic, malignant, or benign. In cases with follow‐up imaging, lesion response was assessed by comparing the tracer uptake and lesion extent on follow‐up imaging with those on baseline imaging to support malignant lesion labeling according to therapy response rather than single‐time‐point expert visual interpretation.

Suspicious findings were identified as having focal tracer uptake that exceeded the surrounding background activity and did not correspond to known physiologic distribution patterns on baseline PET/CT. These findings, identified on baseline ^1^ ^8^F‐PSMA‐1007 PET/CT, were categorized using two reference criteria to investigate lesion discrimination under different levels of diagnostic certainty: imaging follow‐up–based response to therapy (task 1) and baseline expert interpretation by a nuclear medicine physician (task 2). A response to therapy on follow‐up imaging was defined as a visible reduction in lesion size and/or tracer uptake compared with baseline findings. This definition assumed that therapy‐responsive lesions represented true malignant PSMA expression.

In task 1, labels were assigned based on post‐treatment imaging follow‐up (Table 1). Findings from follow‐up PET/CT that demonstrated a response to therapy were classified as malignant (class 1), irrespective of baseline expert interpretation. Findings that were characterized as malignant at baseline but did not respond to therapy or lacked follow‐up data were excluded as it was unclear whether they were truly malignant or false positives.

**TABLE 1:** Findings classification for tasks 1 and 2.

	Task 1			Task 2		
Class	No. before augmentation	No. after augmentation (original + augmented)	No. after filtering (R^2^ > 0.7)	No. before augmentation	No. after augmentation (original + augmented)	No. after filtering (R^2^ > 0.7)
0	49	147(49 + 198)	126	49	147(49 + 198)	126
1	229	229	208	372	229	341
Total	278	376	334	421	376	467

In task 2, lesion labels were assigned solely based on baseline expert interpretation, independent of follow‐up responsiveness, for comparison with the literature (Table 2). Findings interpreted as benign or nonspecific by the physician were classified as benign (class 0), whereas findings interpreted as malignant were classified as malignant (class 1). This task reflected routine clinical decision‐making based on single time‐point PET/CT interpretation and included lesions regardless of follow‐up imaging availability.

**TABLE 2:** Overview of profile‐based features.

Feature	Description
Profile length (*x*, *y*, *z*)	Number of pixels in the trimmed intensity profile along each axis
Mean intensity (*x*, *y*, *z*)	Average intensity along the profile for each axis
Intensity SD (*x*, *y*, *z*)	SD of intensity values for each axis
Max intensity (*x*, *y*, *z*)	Max intensity value along the profile for each axis
Max position (*x*, *y*, *z*)	Location of the max intensity along the profile (mm) for each axis
Min intensity (*x*, *y*, *z*)	Min intensity value along the profile for each axis
Intensity range (*x*, *y*, *z*)	Difference between maximum and minimum intensity values along each profile (max‐min)
Skewness (*x*, *y*, *z*)	Skewness of intensity distribution for each axis
Kurtosis (*x*, *y*, *z*)	Kurtosis of intensity distribution for each axis
Intensity AUC (*x*, *y*, *z*)	Computed using trapezoidal numerical integration for each axis
Mean gradient (*x*, *y*, *z*)	Mean first derivative of the intensity profile for each axis
Gradient SD (*x*, *y*, *z*)	SD of gradient values for each axis
Max gradient (*x*, *y*, *z*)	Max gradient magnitude along the profile for each axis
Min gradient (*x*, *y*, *z*)	Min gradient magnitude along the profile for each axis
Mean skewness (*xyz*)	Mean skewness across *x*‐, *y*‐, and *z*‐axes
Mean intensity (*xyz*)	Average of mean intensities across *x*‐, *y*‐, and *z*‐axes
Intensity SD (*xyz*)	Average of intensity SDs across *x*‐, *y*‐, and *z*‐axes $\left(\frac{SD_{x}+SD_{y}+SD_{z}}{3}\right)$
Max intensity ratios (*x*/*y*, *x*/*z*, *y*/*z*)	Ratios of max intensity values between axes
Max gradient ratios (*x*/*y*, *x*/*z*, *y*/*z*)	Ratios of max gradient values between axes

Abbreviations: AUC, area under the curve; max, maximum; min, minimum; SD, standard deviation.

### 2.4 CAD system

The developed CAD system comprised three distinct feature groups that were evaluated in a classifier‐based framework. These features were extracted from one‐dimensional intensity profiles extending along each of the three orthogonal spatial directions through a point in the lesion (x, y, and z). The first feature group consisted of descriptors extracted directly from the intensity profiles. The second group comprised features derived from the Gaussian fitting of these profiles. The third group consisted of the raw intensity values of the profiles for each spatial direction, which were used as direct inputs. The first and second feature groups were provided as inputs to an RF classifier, whereas the third feature group was provided as inputs to an MLP classifier. The outputs of these base classifiers were subsequently combined and fed into a logistic regression model, which performed the final differential diagnosis.

### 2.5 Pre‐processing

Suspicious findings were manually contoured on PET images using the LifeX software (version 25.06.1) to ensure that extracted features reflected only the signal from each finding.^20^ Each segmented volume of interest was exported in RTSTRUCT Digital Imaging and Communications in Medicine (DICOM) format. For subsequent analysis, the RTSTRUCT files were matched to the corresponding PET image series using DICOM metadata. PET images were loaded slice by slice and sorted according to their spatial position along the axial direction. For each finding, an RTSTRUCT‐defined binary mask was applied to the corresponding PET volume, and voxel intensities outside the segmented volume of interest were set to zero. This procedure isolated the image region corresponding to each finding from the surrounding background while preserving its original spatial geometry and acquisition metadata. The masked volumes were saved as derived DICOM series, retaining only the slices containing nonzero voxels.

Data augmentation techniques were used to address the imbalance between benign and malignant findings. Before profile and feature extraction, the Medical Open Network for AI framework (version 1.5.1)^21^ was used to generate augmented images for the patients with benign findings. Specifically, the applied spatial variations included random rotations of up to 0.1 radians across all three axes, random spatial translations of up to 5 pixels in the axial plane (*x*‐ and *y*‐axes), and random scaling variations of up to 4% in the axial plane. Bilinear interpolation with border padding was used to maintain anatomic continuity and prevent edge artifacts. For each original volume, two augmented versions were generated and filtered as described in detail in the “Gaussian‐fit–derived feature extraction” subsection of the Methods section (Table 1).

**FIGURE 1:**
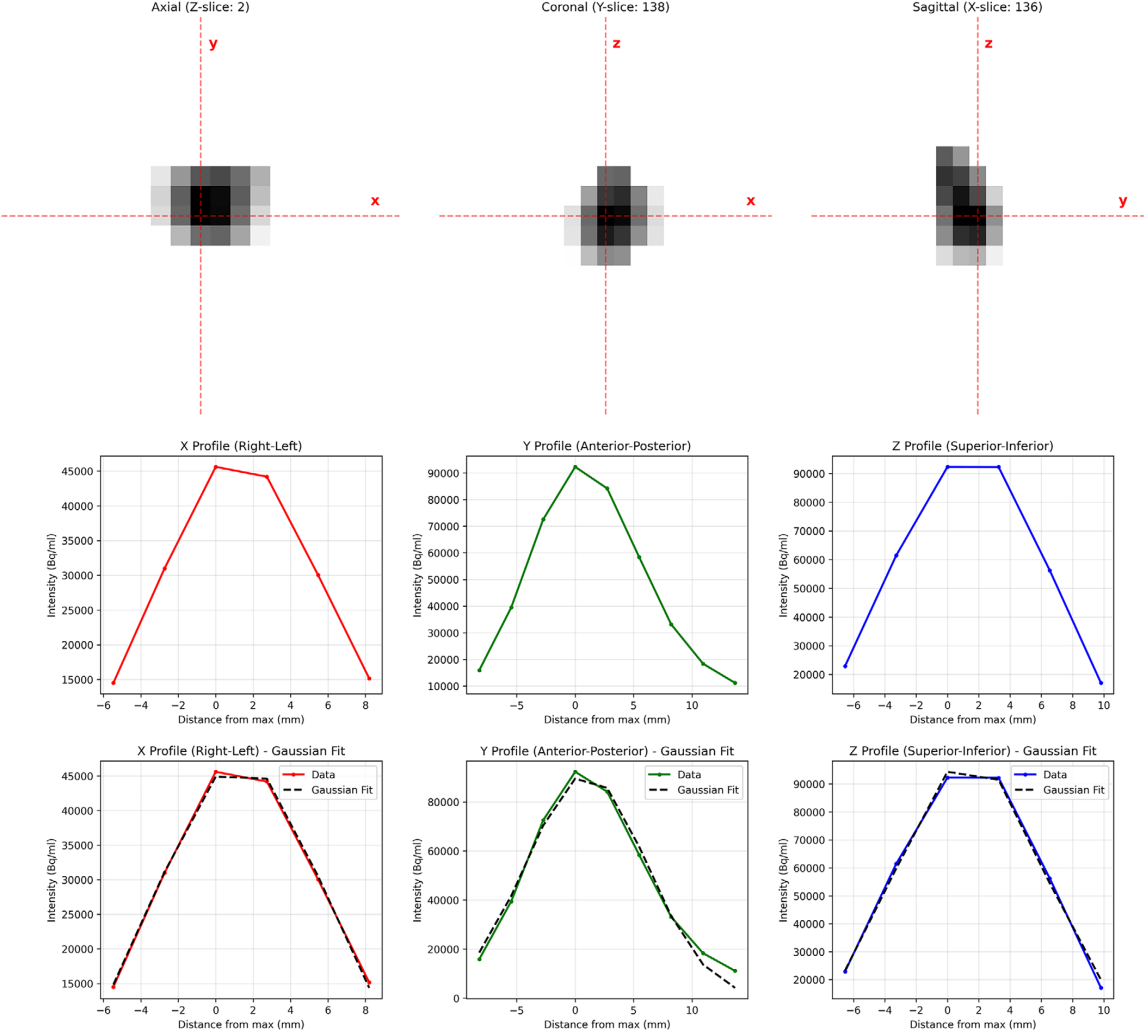
Visualization of the 1‐dimensional intensity profile extraction and Gaussian fitting process for a representative finding. Top row: Axial, coronal, and sagittal cross‐sections of the finding; the red dashed crosshairs intersect at the maximum intensity voxel. Middle row: Extracted raw intensity profiles along the *x*‐ (right‐left), *y*‐ (anterior‐posterior), and *z*‐ (superior‐inferior) axes. The horizontal axis represents the physical distance (mm) from the maximum intensity peak, computed using the corresponding voxel spacing. Bottom row: The extracted intensity profiles (solid lines) overlaid with their respective Gaussian fits (dashed black lines). max, maximum.

For each finding in the combined dataset (original + augmented images), the maximum‐intensity voxel was identified and used as the reference point for the profile extraction of each segmented finding. One‐dimensional intensity profiles were extracted along the three orthogonal axes (*x*, *y*, and *z*) passing through the maximum‐intensity voxel. The *x*‐ and *y*‐axes were defined within the axial plane and corresponded to the left‐right and anterior‐posterior directions, respectively, and the *z*‐axis corresponded to the superior‐inferior direction (Figure 1). To remove noninformative background, each extracted profile was trimmed by removing consecutive zero‐valued samples at both ends, retaining only the contiguous nonzero samples corresponding to the segmented finding. Due to variability in finding size within and across patients, intensity‐profile lengths varied according to its spatial extent.

### 2.6 Feature design overview

A feature‐extraction approach was used to classify malignant and benign ^1^ ^8^F‐PSMA findings on baseline PET imaging. Rather than relying exclusively on voxel‐wise radiomics or end‐to‐end deep‐learning representations, we extracted one‐dimensional intensity profiles centered on the maximum‐uptake voxel for each finding. From these profiles, we derived two sets of features. Profile‐based features were designed to describe the distribution, extent, and heterogeneity of the uptake along each axis, and Gaussian‐fit‐based features provided a parametric and physically interpretable characterization of the profile shape, peak sharpness, and spatial spread. This broad feature design aimed to capture multiple aspects of lesion appearance and to enable the systematic evaluation of which intensity profile characteristics carry information that can be used to differentiate benign from malignant findings. Subsequent feature selection and ensemble modeling were used to identify and combine the most discriminative features.

### 2.7 Profile‐based feature extraction

For each trimmed intensity profile, a set of descriptive features was computed to quantify its intensity distribution, peak characteristics, and spatial heterogeneity. For each profile, the profile length in pixels was recorded. First‐order statistical features—including means and standard deviations, minimum and maximum intensity, skewness, and kurtosis—were extracted. The intensity range for each axis and the position of the maximum‐intensity value along the profile were expressed in mm.

Profile samples were subsequently indexed in physical distance (mm) using the voxel spacing of the corresponding axis (2.73 mm for the *x*‐ and *y*‐axes and 3.27 mm for the *z*‐axis). The area under the intensity‐profile curve was computed using numerical integration with respect to physical distance, providing a measure of the total extent of the signal.

Furthermore, gradient‐based features were obtained using the first derivative of the intensity profiles to quantify local intensity transitions and the spatial heterogeneity along each axis. The mean and standard deviation, maximum, and minimum were calculated for the gradient of each profile. Additionally, cross‐axis features were computed by combining profile‐derived metrics across the three axes. These features included the ratios of maximum intensity between axes and ratios of maximum gradient between axes.

Finally, summary features across the three axes were computed to characterize the overall intensity behavior of each finding. These features included the average of the mean intensity values and the corresponding standard deviations and the mean skewness across all three axes. An overview of all extracted profile‐based features is provided in Table 2.

### 2.8 Gaussian‐fit–derived feature extraction

Each intensity profile was fitted using a Gaussian function to characterize the peak shape and spatial spread. For each axis, a Gaussian model of the form

(1) $$y=\alpha \cdot e^{-\;\frac{{\left(x-\mu \right)}^{2}}{2\sigma^{2}}}\;\;$$

was fitted to the intensity profile using nonlinear least squares, where y denotes the modeled profile intensity, x denotes the distance along the profile, α denotes the peak amplitude, μ denotes the location of the peak, and σ denotes the standard deviation describing the spatial spread of the profile. The distance x and the fitted parameters μ and σ were expressed in mm using the corresponding voxel spacing. To standardize the fitting across findings, the distance axis was recentered such that x=0 corresponded to the maximum‐intensity voxel. Thus, μ represented an offset relative to the maximum‐intensity voxel. The coefficient of determination (*R* ^2^) was computed both as a feature and as a measure of fit quality.

Additional Gaussian‐derived features were computed from the fitted parameters (α,μ,σ) for each axis. These features included the full width at half maximum (FWHM), estimates of peak sharpness, and maximum slope. To summarize three‐dimensional behavior, the mean FWHM across axes and FWHM ratios (*x*/*y*, *x*/*z*, and *y*/*z*) were computed. In addition, the Gaussian spread volume, axis‐wise peak sharpness, and maximum slope were calculated. Slope ratios (*x*/*y*, *x*/*z*, and *y*/*z*) and the three‐dimensional gradient magnitude were also computed. Findings were only retained if the Gaussian fit achieved a coefficient of determination *R* ^2^ of at least 0.7 across all three orthogonal intensity profiles (*x*‐, *y*‐, and *z*‐axes), consistent with the established benchmarks for strong model fit in medical imaging.^22^ Findings that did not meet this filtering criterion were excluded from the model training and performance evaluation to ensure the reliability of the Gaussian‐derived feature set (Table 1). An overview of all extracted Gaussian‐based features is provided in Table 3.

**TABLE 3:** Overview of Gaussian‐based features.

Feature	Description
Amplitude (*x*, *y*, *z*)	Fitted Gaussian amplitude (α), representing the peak intensity of the fitted profile along each axis
Gaussian peak position (*x*, *y*, *z*)	Fitted Gaussian mean (μ) (mm), representing the spatial offset of the peak relative to the max‐intensity voxel after re‐centering (*x* = 0)
Gaussian sigma (*x*, *y*, *z*)	Standard deviation (σ) (mm) of the fitted Gaussian
Gaussian fit profile length (*x*, *y*, *z*)	Number of profile samples used for Gaussian fitting along each axis
*R* ^2^ (*x*, *y*, *z*)	Coefficient of determination
Gaussian max slope (*x*, *y*, *z*)	Max slope of the fitted Gaussian profile along each axis; computed as $\frac{\alpha }{\sigma \cdot e}$ (intensity/mm)
FWHM (*x*, *y*, *z*)	Computed as FWHM=2.35482·σ
Mean FWHM	Mean FWHM value averaged across *x*‐, *y*‐, and *z*‐axes
FWHM ratio (*x*/*y*, *x*/*z*, *y*/*z*)	Ratios of FWHM values between axes
Gaussian spread volume	3D Gaussian spread volume, computed as $\alpha_{mean}{\left(2\pi \right)}^{3/2}\sigma_{x}\sigma_{y}\sigma_{z}$, where $$\alpha_{\mathrm{mean}}=\frac{\alpha_{x}+\alpha_{y}+\alpha_{z}}{3}$$
Peak sharpness	Computed as $\frac{1}{mean(\sigma_{x},\;\sigma_{y},\;\sigma_{z})}$
Gaussian max slope ratio (*x*/*y*, *x*/*z*, *y*/*z*)	Ratios of Gaussian‐derived max slopes between axes
3D gradient magnitude	Computed as $\sqrt{{\left(max\;slope_{x}\right)}^{2}+{\left(max\;slope_{y}\right)}^{2}+{\left(max\;slope_{z}\right)}^{2}}$

Abbreviations: 3D, 3‐dimensional; max, maximum.

### 2.9 Stacking ensemble architecture

A stacking ensemble model was developed. This architecture was chosen for its superior performance compared with single‐model approaches^23^, ^24^ such as bagging and boosting, as demonstrated by Mahajan et al.^25^

**FIGURE 2:**
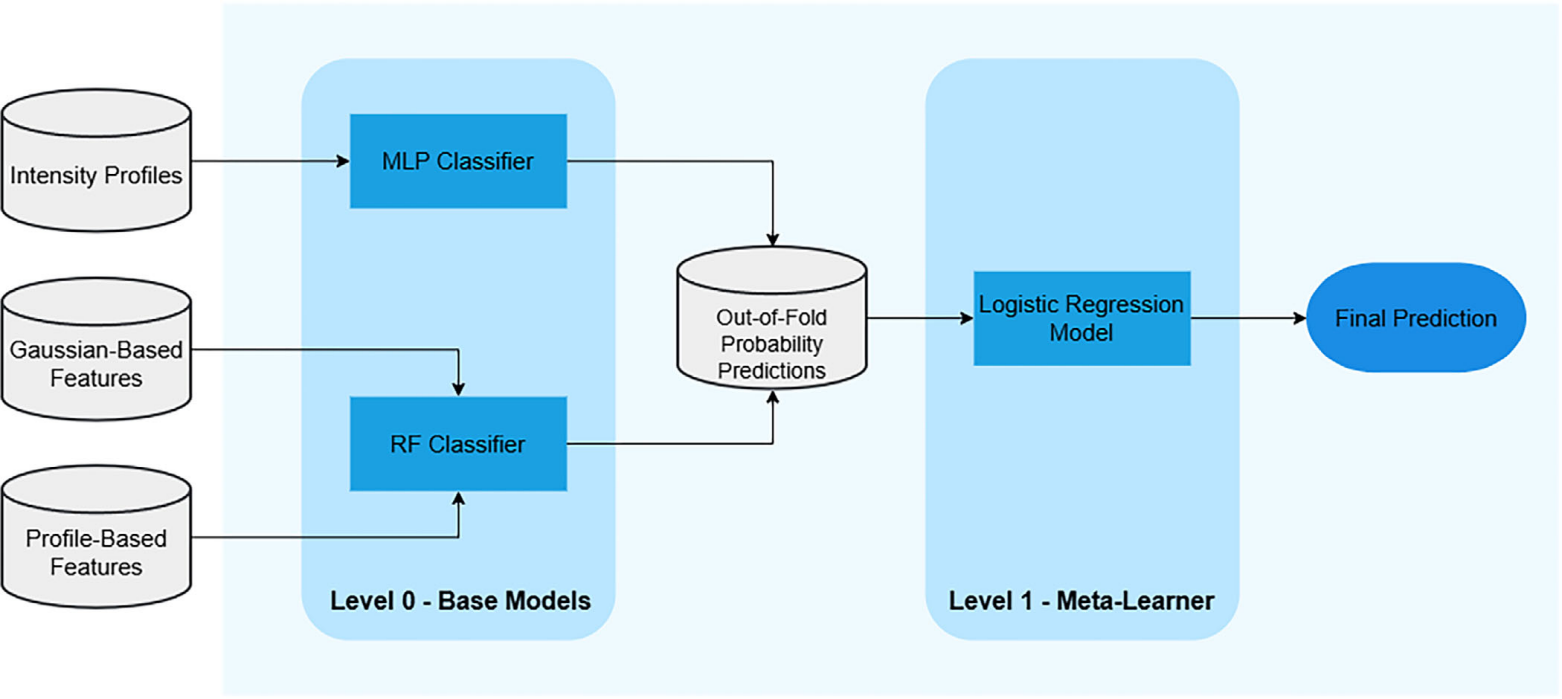
Overview of the customized stacking ensemble architecture combining intensity profiles and derived features using multilayer perception (MLP) and random forest (RF) base learners and a logistic regression meta‐learner.

The stacking ensemble model comprised two layers: level‐0 base learners and a level‐1 meta‐learner, designed to combine different representations of the extracted PET‐derived information. At level 0, two base learners were used: an MLP and an RF classifier. The probabilistic outputs of these level‐0 models were subsequently used as input to a logistic regression meta‐learner at level 1 (Figure 2).

At level 0, the MLP and the RF classifiers were trained in parallel. For each finding, the intensity profiles extracted along the *x*‐, *y*‐, and *z*‐axes were provided as inputs to the MLP classifier. The MLP architecture incorporated two fully connected hidden layers with 128 and 64 neurons, respectively, using ReLU activation functions. Training was performed using the Adam optimizer with an initial learning rate of 0.001. The maximum number of training iterations was set to 600, and early stopping was used to prevent overfitting. The batch size was set to “auto,” which in scikit‐learn sets the batch size to 200 or the number of available samples, whichever is smaller. Since the model was trained within the cross‐validation procedure, the number of samples refers to the training samples available in each training fold rather than the total dataset size. In our study, because the training folds consistently exceeded 200 samples, this resulted in an effective mini‐batch size of 200. Profile‐ and Gaussian‐based features were fed into the RF classifier, which was configured with 300 trees and no predefined maximum tree depth. Balanced subsampling class weights were applied to address class imbalance between the malignant and benign findings. The RF base model was trained on a subset of features that were selected with a feature‐importance analysis method, the forward‐feature selection. Forward‐feature selection was applied within each training fold using an RF estimator and internal cross‐validation, optimizing classification accuracy. A maximum of 15 features was selected per fold. To evaluate the contribution of each selected feature in the RF base model, we analyzed feature‐importance scores and computed the mean and standard deviation importance across all folds. We also tracked the selection rate of each feature.

Model training and evaluation were performed using stratified 10‐fold cross‐validation. In each fold, 90% of the findings were assigned to the training set and used for model development, including preprocessing, forward‐feature selection, training of the MLP and RF base learners, and training of the logistic regression meta‐learner. The remaining 10% of the findings were held out as an independent test set and were used only for final performance evaluation. Within each training set, five‐fold internal cross‐validation was applied for forward‐feature selection of the RF input features and for generating out‐of‐fold probability predictions from the MLP and RF base learners. In each internal split, four folds were used for base‐learner training and one fold served as a validation set for generating out‐of‐fold probability predictions from the MLP and RF base learners. These out‐of‐fold predictions were used to train the level‐1 logistic regression meta‐learner.

The meta‐learner was a logistic regression classifier that combined the probability outputs of the MLP and RF base models to generate the final predicted diagnosis. Model performance was evaluated on the test set using standard classification metrics, including accuracy, sensitivity, specificity, F1‐score, and AUROC.

The final stacking ensemble configuration, which combined the MLP and RF classifiers as base learners with a logistic regression meta‐learner, was selected after evaluating several alternative architectures. Various base‐learner combinations were evaluated, including more complex architectures that used separate RF classifiers for the Gaussian‐ and profile‐derived feature sets alongside the MLP. However, integrating all engineered features into a single RF classifier achieved similar performance while significantly reducing overall model complexity.

All pre‐processing, feature extraction, and classification were conducted on a personal computer equipped with an Intel Core i9‐10850K central process unit and 64 GB random access memory, running Ubuntu 20.04.6 LTS and Python 3.13.2.

## 3 RESULTS

To evaluate findings’ characterization on ^18^F‐PSMA‐1007 PET/CT imaging, the extracted intensity‐profile–based features were evaluated within the stacking ensemble framework under 2 binary classification schemes: imaging follow‐up–based response to therapy and baseline expert‐based interpretation. Performance was assessed using stratified 10‐fold cross‐validation at the lesion level. The mean classification metrics across folds for the augmented datasets are summarized in Table 4, and the corresponding accuracies and confusion matrices are presented in Table 5.

**TABLE 4:** Performance metrics of the stacking ensemble model for follow‐up–based (task 1) and expert‐based (task 2) labeling.

Classification task	Accuracy^a^	Sensitivity^a^	Specificity^a^	AUROC^a^
Task 1 (follow‐up–based)	92.5% (SD, 3.5%)	92.3% (SD, 4.6%)	93.0% (SD, 9.2%)	0.97
Task 2 (expert–based)	91.7% (SD, 5.2%)	93.8% (SD, 4.5%)	85.8% (SD, 8.6%)	0.97

Abbreviation: AUROC, area under the receiver operating characteristic curve.

^a^ Values are reported as mean (SD) across 10‐fold cross‐validation.

**TABLE 5:** Confusion matrix of the stacking ensemble model in task 1 (follow‐up‐based classification) and task 2 (expert‐based classification).

	Task 1, no.			Task 2, no.		
Class	Predicted benign	Predicted malignant	Overall accuracy	Predicted benign	Predicted malignant	Overall accuracy
True benign	117	9		108	18	
True malignant	16	192		21	320	
Predictive Value	(NPV) 88.0%	(PPV) 95.5%		(NPV) 83.7%	(PPV) 94.7%	
Overall accuracy			92.5%			91.7%

Abbreviations: NPV, negative predictive value; PPV, positive predictive value.

For the follow‐up‐based classification (334 lesions; 126 benign and 208 malignant lesions after augmentation and filtering), the stacking ensemble model yielded a mean accuracy rate of 92.5% (SD, 3.5%) and sensitivity and specificity rates of 92.3% (SD, 4.6%) and 93.0% (SD, 9.2%), respectively. The mean F1 score was 93.9% (SD, 2.8%), and the AUROC was 0.97. The aggregated confusion matrix showed 192 true positives, 117 true negatives, 16 false negatives, and 9 false positives, with corresponding positive predictive value (PPV) and negative predictive value (NPV) of 95.5% and 88.0%, respectively (Table 5).

For the baseline expert‐based classification (467 lesions; 126 benign and 341 malignant lesions after augmentation and filtering), the stacking ensemble model achieved a mean accuracy rate of 91.7% (SD, 5.2%) and sensitivity and specificity rates of 93.8% (SD, 4.5%) and 85.8% (SD, 8.6%), respectively. The mean F1 score was 94.2% (SD, 3.6%), and the AUROC was 0.97. The aggregated confusion matrix demonstrated 320 true positives, 108 true negatives, 21 false negatives, and 18 false positives, with corresponding PPV and NPV of 94.7% and 83.7%, respectively (Table 5).

**FIGURE 3:**
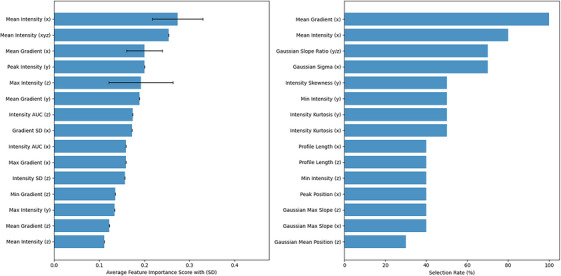
Top 15 features selected by forward‐feature selection for task 1 (follow‐up–based classification). Left: mean feature importance with SD (black straight lines at the top of bars) across 10‐fold lesion‐level cross‐validation. Right: feature selection frequency expressed as percentage of folds in which each feature was selected. AUC, area under the curve; Max, maximum; Min, minimum.

**FIGURE 4:**
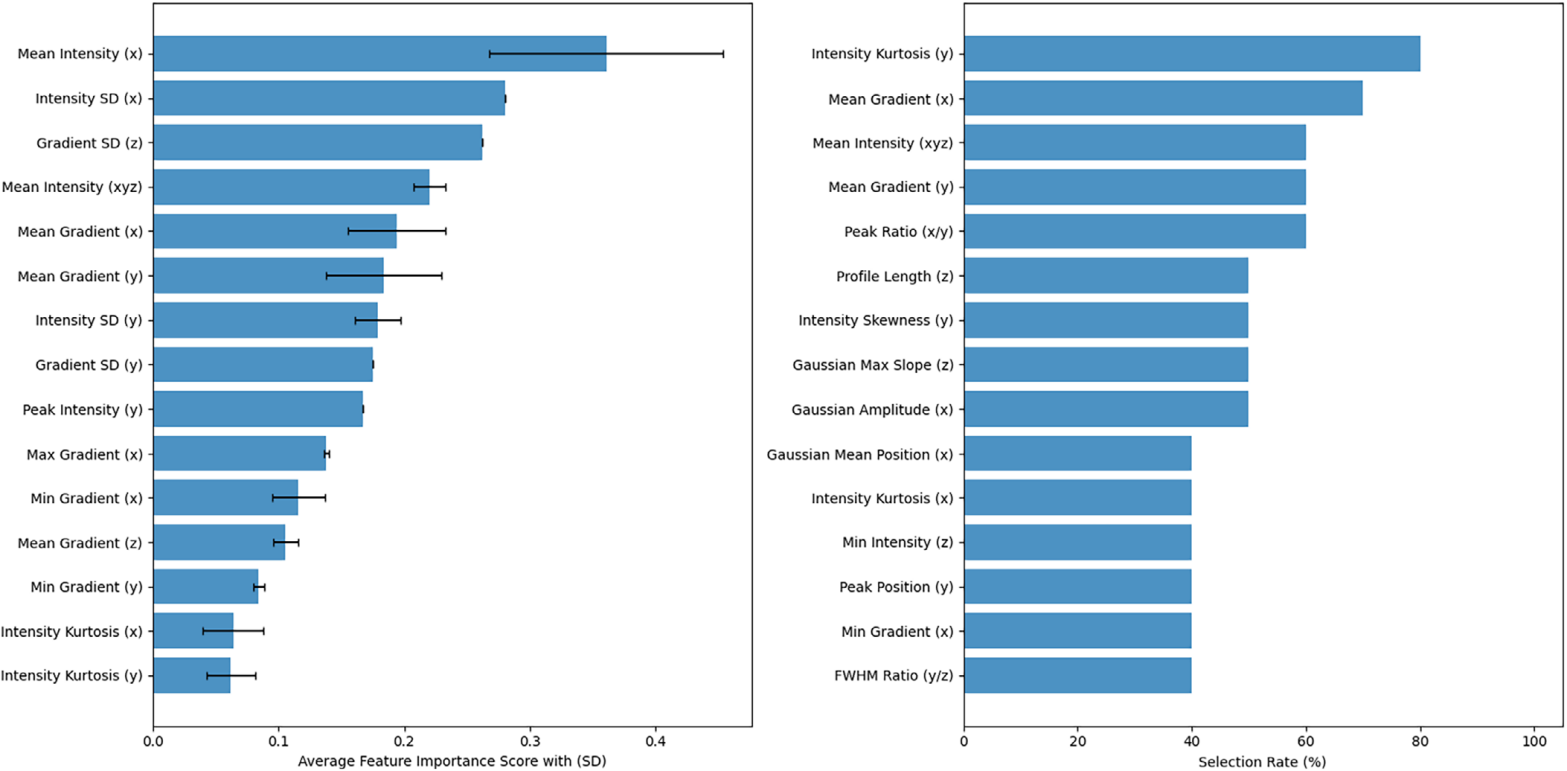
Top 15 features selected by forward‐feature selection for task 2 (expert‐based classification). Left: mean feature importance with SD (black straight lines at the top of bars) across 10‐fold lesion‐level cross‐validation. Right: feature selection frequency expressed as percentage of folds in which each feature was selected. AUC, area under the curve; Max, maximum; Min, minimum.

After feature importance analysis, the top 15 features ranked by mean importance score, along with their corresponding selection rates, for tasks 1 and 2 are illustrated in Figures 3 and 4, respectively. In Task 1 (Figure 3), mean intensity (*x*) had the highest average feature importance score, followed by mean intensity (*xyz*) and mean gradient (*x*). Regarding selection rates, mean gradient (*x*) was the only feature selected in 100% of the training folds. Mean intensity (*x*) followed with an 80% selection rate, while the top Gaussian‐derived features, Gaussian slope ratio (*y*/*z*) and Gaussian sigma (*x*), were selected in 70% of the folds. For Task 2 (Figure 4), mean intensity (*x*) again achieved the highest average importance score. The subsequent highest‐ranking features for this task were the intensity sd (*x*) and gradient sd (*z*). The selection rate distribution for Task 2 differed from Task 1, with no feature reaching a 100% selection rate. Instead, intensity kurtosis (*y*) was the most frequently selected feature (80%), followed by mean gradient (*x*) (70%).

## 4 DISCUSSION

The present study proposed a computer‐aided classification framework for the differential diagnosis of ^1^ ^8^F‐PSMA‐1007 PET/CT findings in patients with PCa and BCR. We used post‐therapy imaging follow‐up as a reference standard for identifying malignant findings, aiming to provide a more reliable labeling strategy than baseline expert interpretation alone. In addition, to our knowledge, quantitative descriptors extracted from one‐dimensional intensity profiles centered on the maximum uptake voxel of each finding and both profile‐based and Gaussian‐derived features were integrated for the first time within a stacking ensemble classifier. The proposed framework achieved high classification performance under both tasks. The follow‐up‐based and expert‐based classification tasks both performed well, achieving accuracy rates greater than 90% and sensitivity and specificity rates above 85%. Both tasks had an AUROC of 0.97.

Feature‐importance analysis across the folds indicated that a combination of intensity distribution, spatial heterogeneity, and parametric peak‐shape characteristics led to differentiation between the tasks. Feature importance and selection rate analysis (Figures 3 and 4) revealed distinct quantitative trends between the 2 classification tasks. Overall, the introduced profile‐based descriptors demonstrated higher predictive value and selection frequency than the Gaussian‐derived features in both models. In the follow‐up–based classification (task 1), intensity magnitude and spatial gradients were the primary drivers of model performance. Specifically, mean intensity (*x*) and mean intensity (*xyz*) had the highest mean feature importance. However, mean gradient (*x*) was the most consistently robust descriptor in task 1, achieving a 100% selection rate across all training iterations, suggesting that localized spatial changes were consistently important for identifying therapy‐response outcomes. In detail, class 1 (true malignant) findings demonstrated a substantially higher average mean gradient (*x*) (971.48 ± 857.70) compared to class 0 (benign) findings (153.08 ± 183.11). As the mean first derivative of the intensity profile, the mean gradient mathematically captures the steepness of intensity transitions across a finding. In the context of PSMA PET imaging, the substantially higher mean gradient in class 1 translates to sharper, more abrupt changes in PSMA concentration within the finding itself. This suggests that findings exhibiting a verified therapy response are characterized by more focal or heterogeneous uptake patterns. Conversely, the lower mean gradient in class 0 reflects smoother, more uniform internal uptake transitions. This pattern is also observed in task 2, where mean gradient (*x*) has a selection rate of 70%. In detail, class 1 (malignant) findings demonstrated also a substantially higher average mean gradient (*x*) (948.30 ± 842.73) compared to class 0 (benign) findings (153.08 ± 183.11). However, given the overlap in variability between the two classes, interpretations and conclusions should be considered exploratory. Select Gaussian‐derived metrics, such as Gaussian slope ratio (*y*/*z*) and Gaussian sigma (*x*), also maintained high selection frequencies (70%) in task 1, indicating that parametric shape descriptors played a supporting role.

The expert‐based classification (task 2) more strongly relied on primary intensity features and distribution heterogeneity than task 1. In this task, mean intensity (*x*) was the feature with the highest mean importance, followed closely by variance measures such as intensity standard deviation (*x*) and gradient standard deviation (*z*). Distribution shape feature group was the most selected in task 2, with intensity kurtosis (*y*) emerging as the most frequently selected feature (80% selection rate). Quantitatively, class 1 (malignant) findings demonstrated a higher mean kurtosis (−0.695 ± 0.558) compared to class 0 (benign) findings (−0.912 ± 0.721). In profile analysis, kurtosis measures the weight of the tails of the intensity distribution relative to a normal distribution. The lower kurtosis value in class 0 suggests lighter tails, corresponding to a more uniform distribution of PSMA uptake without extreme intensity variations. In contrast, the higher kurtosis values in class 1 indicate heavier tails, potentially reflecting more extreme intensity values. This is consistent with the visual interpretation of malignant PSMA‐avid findings as more localized foci of increased tracer uptake, whereas benign or nonspecific findings may exhibit less sharply concentrated uptake patterns. In task 1, kurtosis (*y*) has a selection rate of 50%, showing that it is a less important descriptor in benign versus true malignant differentiation. However, given the overlap in variability between the two classes, interpretations and conclusions should be considered exploratory. Despite these differences, core profile‐based metrics such as mean intensity (*x*) and mean gradient (*x*) were prominent across both tasks. These findings suggest that raw tracer uptake and its spatial gradient along the *x*‐axis were robust and reproducible descriptors, regardless of the reference standard used.

The present results are consistent with findings from recent efforts to integrate AI into PSMA PET/CT analysis. Several studies have reported approaches with high classification performance in differentiating malignant from benign PSMA uptake. For example, Erle et al.,^15^ Li et al.,^16^ and Moazemi et al.^17^ reported AUROC values ranging from 0.97 to 0.98 using machine‐learning or deep‐learning frameworks. However, these studies rely on baseline expert interpretation as the reference standard; thus, trained models effectively learn to reproduce expert labeling rather than confirm actual disease status. Thus, these models’ classification performance may be inflated by inter‐reader variability and may not reflect their true diagnostic accuracy.

A principal methodologic contribution of the present study is the use of post‐therapy imaging follow‐up as a reference standard. In clinical practice, histopathologic confirmation of PSMA‐avid lesions in patients with BCR is often not feasible, particularly for small lymph nodes or distant metastases.^26^ Consequently, treatment response observed on follow‐up imaging provides an objective indicator of true malignant PSMA expression complementary to expert visual interpretation. One follow‐up–based approach was explored by Zhao et al.,^18^ who used pathologic or clinical follow‐up as a reference standard to train a deep‐learning model on combined PET and CT data, achieving an AUROC of 0.79 and an accuracy rate of 78%.^18^ In comparison, our follow‐up‐based classification (task 1) achieved an AUROC of 0.97 and an accuracy rate of 92.5%, with a corresponding sensitivity rate of 92.3% and specificity rate of 93.0%. Although differences in datasets and study design may account for these discrepancies, the strong performance observed in our study suggests that intensity‐profile–derived descriptors, combined with our proposed stacking ensemble model, offer a more robust characterization of malignant PSMA uptake than similar approaches.

Notably, low specificity has been reported in some cases in which PET features alone were used; for example, Moazemi et al.^17^ reported a specificity of 67% and Erle et al.^15^ reported a specificity of 83%. However, our expert‐based classification (task 2), which incorporated profile‐based feature, achieved a specificity of 85.8% and an AUROC of 0.97. These findings further support that approaches including profile‐based features have fewer false‐positive classifications than solely expert‐labeled approaches.

Several AI‐based approaches have been proposed for PSMA PET/CT analysis, including lesion‐level classification, patient‐level prediction, and PSMA‐RADS–based categorization schemes.^15^, ^16^, ^27^, ^28^ These approaches have been associated with varying performance metrics: Accuracies have ranged from 76.4% to 94.5%, and AUROC values have ranged between 0.851 and 0.98.^15^, ^16^, ^27^, ^28^ However, a direct head‐to‐head comparison between these studies and our proposed framework is not feasible owing to fundamental methodologic differences, including variations in the defined classification tasks.

The present study introduced new methodologic criteria for ground‐truth selection in malignancy classification and used intensity‐profile–based features from each segmented finding as input for a powerful supervised classification model. The proposed scheme achieved equivalent or superior performance to methods reported in related studies^15^, ^16^, ^17^, ^18^ under both expert‐labeled and follow‐up‐labeled training conditions.

Despite the strong predictive performance of the proposed framework, several limitations of this study should be acknowledged. The ground truth used in task 1 was based on asymmetric class criteria. Malignant lesions were confirmed through their visible response to therapy on follow‐up imaging, whereas benign lesions lacked an equivalent level of objective verification. An inherent limitation of this imaging‐based approach is the clinical reality of non‐responding malignant lesions despite therapy. To mitigate the risk of misclassifying these non‐responding suspicious findings as benign or malignant, our methodology required that findings without evidence of therapy response, or without follow‐up data altogether, were only assigned to the benign class if they already had a baseline expert interpretation as benign or non‐specific. A larger study with more benign findings confirmed by follow‐up imaging could better characterize this asymmetry and more fully demonstrate the potential of the proposed method. Also, the original clinical dataset exhibited notable size imbalance between the malignant and benign classes, reflecting the clinical reality that elevated PSMA findings in patients with BCR more often indicate malignant PSMA uptake than benign or nonspecific uptake.^5^ To address this imbalance, data augmentation techniques were applied to balance the training sets and mitigate classifier bias. An additional limitation of the present study is that cross‐validation was performed at the lesion level rather than the patient level. Because multiple lesions from the same patient may appear in both the training and test sets within a single fold, the model was potentially exposed to shared patient‐specific characteristics (scanner noise patterns, patient anatomy, and clinical context) during training, which may have led to optimistically biased performance estimates. This concern is partly attenuated in the present study by the single‐center, single‐scanner design, which limits between‐patient variability in acquisition‐related features. Nevertheless, patient‐level data partitioning would provide a more conservative and externally valid estimate of model performance, and future studies should adopt patient‐level cross‐validation or an independent patient cohort for external validation, particularly when extending the framework to multi‐center settings. Future prospective studies with larger, balanced cohorts and histopathologically verified ground truths would further strengthen the clinical applicability of the proposed framework.

## 5 CONCLUSION

Here, we proposed a classification model for the differentiation of suspicious ^1^ ^8^F‐PSMA PET/CT findings grounded in a more reliable follow‐up‐based reference standard and combined with newly introduced intensity‐profile–based descriptors and a customized stacking ensemble classifier. The proposed model demonstrated performance equivalent to or exceeding that of similar published approaches. The integration of this quantitative, profile‐based tool into routine clinical workflow could improve diagnostic confidence and support personalized management for patients with PCa and BCR.

## CONFLICT OF INTEREST STATEMENT

The authors declare no conflicts of interest.
